# Deciphering the biological effects of acupuncture treatment modulating multiple metabolism pathways

**DOI:** 10.1038/srep19942

**Published:** 2016-02-16

**Authors:** Aihua Zhang, Guangli Yan, Hui Sun, Weiping Cheng, Xiangcai Meng, Li Liu, Ning Xie, Xijun Wang

**Affiliations:** 1 National TCM Key Laboratory of Serum Pharmacochemistry, Sino-US Technology Cooperation Center of Chinmedomics, Research Center of Chinmedomics (State Administration of TCM), Laboratory of Metabolomics, Key Pharmacometabolomics Platform of Chinese Medicines, Department of Pharmaceutical Analysis, Heilongjiang University of Chinese Medicine, Heping Road, Harbin, China

## Abstract

Acupuncture is an alternative therapy that is widely used to treat various diseases. However, detailed biological interpretation of the acupuncture stimulations is limited. We here used metabolomics and proteomics technology, thereby identifying the serum small molecular metabolites into the effect and mechanism pathways of standardized acupuncture treatments at ‘Zusanli’ acupoint which was the most often used acupoint in previous reports. Comprehensive overview of serum metabolic profiles during acupuncture stimulation was investigated. Thirty-four differential metabolites were identified in serum metabolome and associated with ten metabolism pathways. Importantly, we have found that high impact glycerophospholipid metabolism, fatty acid metabolism, ether lipid metabolism were acutely perturbed by acupuncture stimulation. As such, these alterations may be useful to clarify the biological mechanism of acupuncture stimulation. A series of differentially expressed proteins were identified and such effects of acupuncture stimulation were found to play a role in transport, enzymatic activity, signaling pathway or receptor interaction. Pathway analysis further revealed that most of these proteins were found to play a pivotal role in the regulation of multiple metabolism pathways. It demonstrated that the metabolomics coupled with proteomics as a powerful approach for potential applications in understanding the biological effects of acupuncture stimulation.

Acupuncture, which utilizes fine needles to pierce through specific anatomical points (called ‘acupoints’), is an ancient traditional Chinese medicine (TCM) therapy^1^. Therapeutic effects of acupoint stimulation primarily work through 14 principal meridians^2^. Nowadays, this traditional technique has become very popular worldwide as a complementary medicine^3^,^4^. It has been widely used to reduce some symptoms or to treat diseases in clinical practice^5^,^6^,^7^. Human studies have found a physiological basis for acupuncture needling that involves both central and peripheral networks^8^. However, it remains insufficient, we still lack a clear picture of ‘how acupuncture works’ and the challenge is therefore to clarify the true meaning. We have to understand the acupuncture and present it in the light of the 21st century scientific thoughts and experimental evidence.

Omics technologies, including proteomics, and metabolomics, have been successfully applied to screen for biomarkers in biological fluids for diseases. They are key technology that serves as the major driving force for translation of acupuncture medicine revolution into practice^9^. Recent advances in systems biology technology has enabled the discovery of biomarkers, and potentially offered ‘the right therapy for the right patient’^10^. Specifically, high-throughput proteomics and metabolomics have been able to identify potential candidates for the effects of acupuncture^11^,^12^. A large number of differentially expressed proteins have been identified and reported as potential biomarkers for the diagnosis and prognosis of several different diseases. The isobaric tag for relative and absolute quantitation (iTRAQ) technique is one of the most widely used approaches in proteomics^13^. Proteomics may help us better understand the changes of multiple proteins involved in the molecular mechanism of acupuncture. Metabolomics aims to reveal various metabolic characteristics of external or internal perturbations to biological systems by profiling low-molecular-weight metabolites in biological samples^14^. Metabonomics and acupuncturology are of some similar characteristics such as entirety, comprehensiveness and dynamic changes and might be a promising method to investigate the molecular mechanism of acupuncture^15^,^16^. Thus, integrative multi-omics analysis will enable a revolution in our in-depth understanding of acupuncture treatment for a variety of diseases.

Acupuncture is a non-pharmacological therapy in which needles are inserted at specific acupoints of the body^17^. In the context of previous literature, Zhang and co-workers used metabolomics analysis to investigate the saliva metabolite biomarkers for acupuncture treatment at ‘Zusanli’ acupoint as a case study^18^. They have identified 26 differential metabolites and found that the phenylalanine metabolism, alanine, aspartate and glutamate metabolism, d-glutamine and d-glutamate metabolism, and steroid hormone biosynthesis pathways were acutely perturbed. The network construction has led to the integration of metabolites associated with the multiple perturbation pathways. Yan *et al.* used high-throughput urine metabolic profiling dissects the biological property of acupuncture point Zusanli based on long-term treatment. The top canonical pathways include alpha-linolenic acid metabolism, d-glutamine and d-glutamate metabolism, citrate cycle, alanine, aspartate, and glutamate metabolism, and vitamin B6 metabolism pathways^19^. However, acupuncture stimulation modulates metabolism pathways in human serum has yet to be investigated. More work needs to be performed to explore the molecular mechanisms underlying acupuncture and this could lead to novel treatments to maximize the therapeutic benefits of acupuncture. Several studies have reported the Zusanli (also known as ST-36) point is commonly used in human to treat a wide range of diseases including gastrointestinal disorders and others^20^,^21^,^22^. However, its underlying biological mechanism remains poorly understood. We here present a large-scale study in which we combine a quantitative proteomic discovery strategy using iTRAQ and an ultra-performance liquid chromatography/electrospray ionization quadruple time-of-flight mass spectrometry (UPLC/ESI-Q-TOF/MS)-based metabolomics approach to examine whether acupuncture stimulation at the Zusanli acupoint modulates metabolism pathways in human.

## Results

### Trajectory and metabolic profling analysis

**Figure 1:**
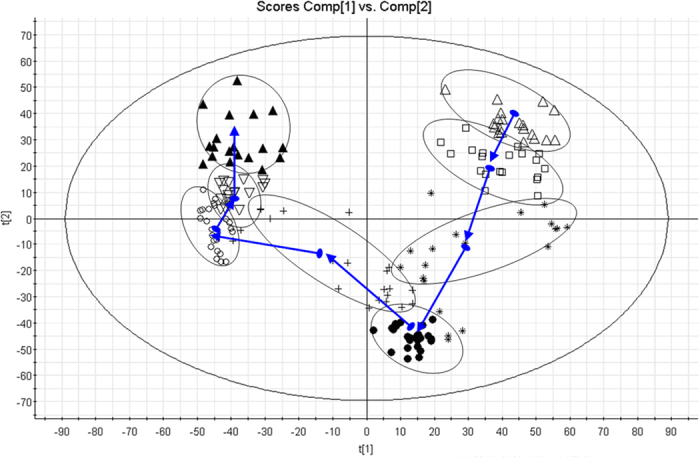
Trajectory analysis of PCA Score plots for the acupuncture treatment. (△: the 1st day; □: the 3rd day; *: the 5th day; ●: the 7th day; +: the 9th day; ○: the 11th day; ▽: the 13th day; ▲: the 14th day).

**Figure 2:**
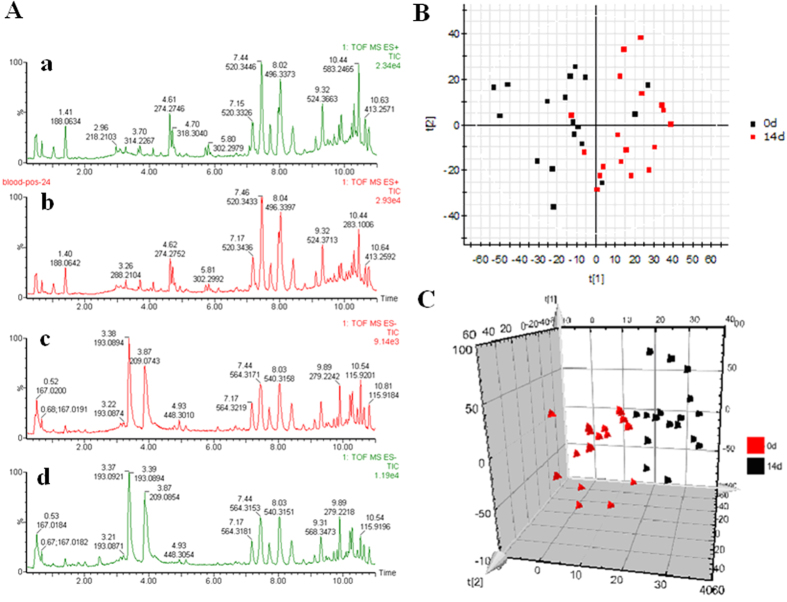
Metabolic profling characterization and pattern recognition analysis. (**A**) UPLC-MS BPI serum chromatograms of acupuncture-treated human in positive mode (a, 0 day; b, 14 day) and negative mode (c, 0 day; d, 14 day); (**B**) PCA score plot for control and acupuncture-treated group in positive mode; (**C**) Trajectory analysis of PCA score plots (3-D) for the serum samples in negative mode.

Typically, the trajectory analysis of principal components analysis (PCA) score plots for the acupuncture treatment can really reflect the differences between the 0 day and the 14th day, and showed metabolic profiles in the different days were separated clearly (Fig. 1). PCA plot of the various group show similar behavior during the early stage of the experiment, but then gradually deviated from one another, on day 14 reached the maximum trend. The tracks of the metabolic profiles also clearly demonstrate the time dependent changes in the urine metabolites. For UPLC/ESI-Q-TOF/MS analysis, aliquots were separated using a Waters Acquity UPLC (Waters, Millford, MA) and analyzed using a quadruple time-of-flight mass spectrometry, which consisted of an electrospray ionization source and linear ion-trap mass analyzer. UPLC-MS BPI serum chromatograms of acupuncture-treated human in positive mode and negative mode showed stable retention time with no drift in all of the peaks, demonstrating the robustness of our analytical method (Fig. 2A). Low molecular mass metabolites could be separated well in the short time of 11 min due to the minor particles (sub-1.7 um). According to the optimized conditions of urine analysis, principal component analysis of quality control samples from liver injury rats was shown in Fig. S1, quality control samples were gathered together to determine during the data collection, demonstrating that the system had excellent stability during the analysis procedure.

### Pattern recognition analysis and screening of the metabolite markers

**Figure 3:**
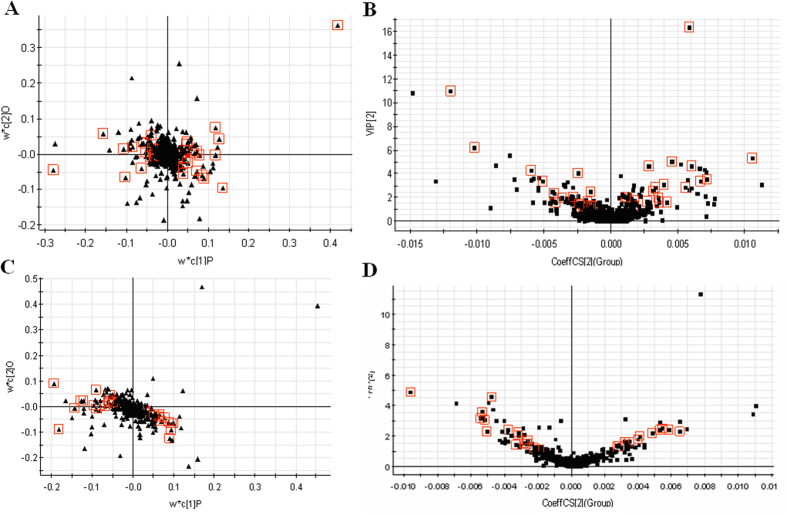
The screening of the metabolite markers. (**A**) PLS loading plot results for 0 day and 14 day in positive mode; (**B**) VIP-plot of OPLS-DA of samples in positive mode; (**C**) PLS loading plot results for 0 day and 14 day in negative mode; (**D**) VIP-plot of OPLS-DA of samples in negative mode.

Both multivariate projection approaches often can be taken, because of their ability to cope with highly multivariate, noisy, collinear and possibly incomplete data. A PLS-DA model was established to evaluate the systemic changes in the metabolome of acupuncture group. The PCA score plots showed that the metabolic profiles of the 0 and 14 day significantly changed as a result of acupuncture-treated (Figs 1C and 2B), indicating that serum biochemical perturbation significantly happened in the acupuncture group. From the corresponding loading plot (Figs 2B and 3A), the variables (ions) far away from the origin contribute significantly to this model. The ions that showed significant difference in abundance between the 0 day and 14 day treated human were contributed to the observed separation and selected from the respective S-plots as potential markers in positive and negative mode (Figs. 2C and 3C). Overall 6197 retention time-exact mass pairs were determined in metabolomic profile of serum samples. To screen the statistically important variables (ions) related to acupuncture, a strategy combining VIP and two-tailed Student’s *t*-test was used. 34 variables with the VIP-value larger than 1.0 and *p-*value less than 0.05 were selected and considered as potential markers representing the metabolic characteristics of acupuncture. Finally, the markers of significant contribution were identified 34 ions (20 in the positive mode, 14 in the negative mode) as differentiating metabolites. From the above plots, various metabolites could be identified as being responsible for the separation between 0 day and 14 day groups, and were therefore viewed as potential biomarkers.

### Identification of important differential metabolites

**Figure 4:**
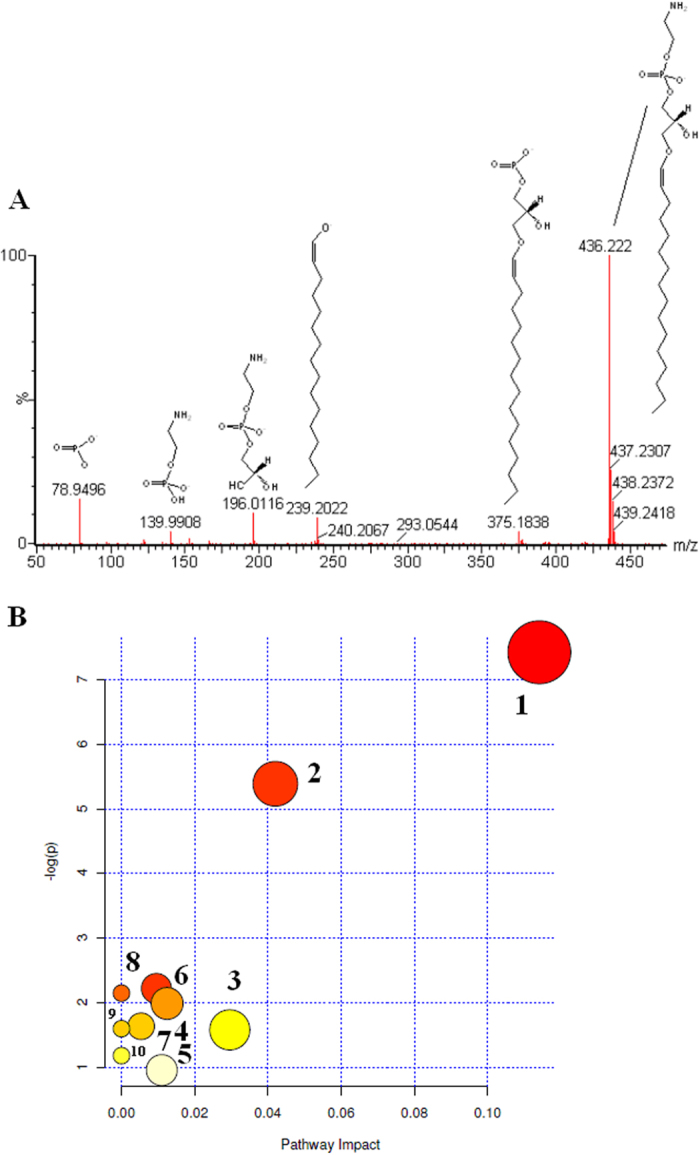
Identification of the important differential metabolites. (**A**) Mass fragment information of LysoPE(P-16:0/0:0) in negative mode. (**B**) Construction of the metabolism pathways in acupuncture-treated human. The map was generated using the reference map by KEGG (http://www.genome.jp/kegg/). The green boxes: enzymatic activities with putative cases of analogy in acupuncture-treated human. 1, glycerophospholipid metabolism; 2, ether lipid metabolism; 3, fatty acid metabolism; 4, glycerolipid metabolism; 5, porphyrin metabolism; 6, sphingolipid metabolism; 7, primary bile acid biosynthesis; 8, fatty acid elongation in mitochondria; 9, fatty acid biosynthesis; 10, tryptophan metabolism.

Robust UPLC/ESI-Q-TOF/MS provides the retention time, precise molecular mass and MS/MS data for the structural identification of metabolites. Structure identification of the significant metabolites was performed according to their molecular ion masses and MS/MS product ion analysis comparing with authentic standards or database resources. The potential elemental composition, degree of unsaturation and fractional isotope abundance of compounds were also obtained. Here we take LysoPE(P-16:0/0:0) in negative mode (with the M-H^−^ molecular ion peak at m/z 436.222) as an example to illustrate our metabolite identification strategy. According to its accurate mass, C_21_H_44_NO_6_P was calculated as the most possible molecular formula by EZinfo 2.0 software. Then, from its tandem MS/MS spectra, the major fragment ions were illustrated in Fig. 4A. Then, the above information was searched and matched in databases of HMDB (http://www.hmdb.ca), METLIN (http://metlin.scripps.edu) and KEGG (http://www.kegg.jp). Finally, this ion of m/z 436.222 was validated by a standard compound and identified as LysoPE(P-16:0/0:0). According to the protocol detailed above, a total of 34 endogenous metabolites were finally identified as markers and listed in Supplementary Table 2.

### Pathway enrichment analysis

Based on the identified metabolite markers, a metabolic pathway analysis facilitating further biological interpretation was performed using MetPA (Metabolomics Pathway Analysis) to reveal the most relevant pathways involved in acupuncture on ST-36. It was shown that metabolite markers were mainly involved in the following pathways: 1, glycerophospholipid metabolism; 2, ether lipid metabolism; 3, fatty acid metabolism; 4, glycerolipid metabolism; 5, porphyrin metabolism; 6, sphingolipid metabolism; 7, primary bile acid biosynthesis metabolism (Fig. 4B and Supplementary Table 3). Acupuncture triggers dynamic changes in metabolic biomarkers were shown in Fig. 4C. The significantly changed metabolites have been found and used to explain the metabolic pathway. Results suggested that these pathways showed the marked perturbations over the entire time-course of acupuncture-treated, and we found that glycerophospholipid metabolism, ether lipid metabolism, fatty acid metabolism (Impact >0.02) were acutely perturbed by acupuncture stimulation.

### Identification of proteome by iTRAQ analysis

To further investigate the effects of acupuncture treatment at ST-36, we used iTRAQ to generate the protein expression profiles. Protein changes of magnitude 1.2-fold or more were considered for further investigation. Using a cutoff of 1.2-fold change and a p-value less than 0.01, total 199 proteins were shown to have significantly different abundance between 0 and 14 days. We found 93 and 106 target proteins were up-regulated and down-regulated, respectively, and that were highly specific to the acupuncture treatment (Supplementary Table 4). It showed the proteins identified, including the name, accession number, score, coverage, molecular weight, PI, and protein expression ratio. Among them, the significance of the 22 proteins with score >1000 would be interesting topic for our future studies.

### Functional analysis of differentially expressed proteins

It is better and easier to understand the function and regulation of the differentially expressed proteins by bioinformatics analysis. The final selected differentially expressed proteins were analyzed using the GO database to determine their molecular function, and participation in biological processes. In the molecular function analysis, most of these proteins were found to play a role in transport, enzymatic activity, signaling pathway or receptor interaction, suggested that the proteins in these functional categories were highly expressed and may be active during the acupuncture treatment condition. These differentially expressed proteins may be related to several metabolic pathways, including carbohydrate digestion and absorption, protein digestion and absorption, amino sugar and nucleotide sugar metabolism, fructose and mannose metabolism, complement and coagulation cascades *etc*. Based on the number of unique proteins identified, the most interesting functional categories were “gastric acid secretion” and “pancreatic secretion”, representing 2.53% and 6.33% of all the protein identified, respectively. The result would facilitate the understanding that ST-36 point is commonly used in human acupuncture to treat gastrointestinal disorders.

Gastric acid is a key factor in normal upper gastrointestinal functions, including protein digestion and calcium and iron absorption, as well as providing some protection against bacterial infections^21^. Stimulation of acid secretion typically involves an initial elevation of intracellular calcium and cAMP, followed by activation of protein kinase cascades, which trigger the translocation of the proton pump, H^+^ -K^+^ -ATPase, from cytoplasmic tubulovesicles to the apical serum membrane and thereby H^+^ secretion into the stomach lumen. The pancreas performs both exocrine and endocrine functions^22^. The exocrine pancreas consists of two parts, the acinar and duct cells. The primary functions of pancreatic acinar cells are to synthesize and secrete digestive enzymes. The major task of pancreatic duct cells is the secretion of fluid and bicarbonate ions (HCO^3−^), which neutralize the acidity of gastric contents that enter the duodenum. An increase in intracellular cAMP by secretin is one of the major signals of pancreatic HCO^3−^ secretion.

To better understand at the proteomic level, the molecular mechanisms and relevant pathways that underlie acupuncture point function, we employed a proteomics strategy to perform functional enrichment analysis, with a special focus on the changes in the biological process after acupuncture treatment. We found 22 target proteins that were highly specific to the acupuncture. The detection of these proteins with distinct regulatory patterns provides evidence that novel biomarkers are actively involved in multifunctional pathways that are likely essential for acupuncture. The biological functions of these critical proteins can be sorted into five groups: (A) generation and degradation of the extracellular matrix, including fibronectin 1, fibrinogen gamma chain isoform CRA_o, and fibrinogen gamma chain isoform CRA_a; (B) the regulation of transcription and translation, as provided by phospholipid transfer protein; (C) acute phase reaction and immunity protection, as provided by immunoglobulin heavy chain constant region mu, immunoglobulin kappa light chain VLJ region, immunoglobulin kappa light chain variable region, Ig gamma3 heavy chain; (D) oxygenation and cell apoptosis, to which anti-pneumococcal antibody 57E2 light chain contributes; and (E) transport and metabolism, as provided by apolipoprotein A-II, apolipoprotein C-II, apolipoprotein B-100 precursor, apolipoprotein B variant, tubulin alpha-4A chain. The characteristic functions of these differentially expressed proteins were enriched within clusters that were based on biological processes such as “immunity”, “cellular apoptosis”, “transport”, “signal transduction”, and “metabolism”. These proteins may therefore be considered candidates for the further investigation of biological mechanism of acupuncture point function. To reveal the associated with signal transduction pathways and/or signaling networks, the differentially expressed proteins were imported into the integrated molecular pathway level analysis (IMPaLA, http://impala.molgen.mpg.de/) tool. It revealed that proteins which were identified together are important for the host response to acupuncture point function.

## Discussion

In this study we present protein and metabolite profiles response to acupuncture treatment in human serum. To identify potential biomarker candidates for acupuncture effects at ST-36, we performed several data analysis, including multivariate pattern recognition analysis, univariate statistical analysis, pathway analysis, and network analysis. We used iTRAQ and UPLC/ESI-Q-TOF/MS approach to identify, quantify, and validate changes in protein and metabolite abundance, also to determine the metabolic disturbances pathways perturbation of ST-36 as a case study. Metabolomic analysis of key regulatory metabolites in acupuncture-treated man was successfully investigated by UPLC/ESI-Q-TOF/MS combined with multivariate statistical analysis. Interestingly, we have identified 34 metabolites relevant for ST-36 between blood samples at 0 day (control) and 14 day (treatment). Of note, we found that acupuncture activated an array of factors. Proteomics holds great promise in contributing to the prevention and cure of disease because it provides unique tools for high-throughput screening of biomarkers and therapeutic targets^23^,^24^,^25^,^26^,^27^. During the acupuncture treatment, molecular function analysis showed that the acupuncture was found to play a role on ST-36 in transport, signaling pathway and receptor interaction etc. Integrated network analysis of the differentially expressed proteins in acupuncture-perturbed yields highly related signaling networks, suggests strongly that the involvement of these signaling networks could be essential for the biological basis of ST-36.

In summary, we have firstly constructed the metabolomic feature profiling and protein networks of ST-36 by using systems biology approach and bioinformatic functional analysis, to realize the full potential of acupuncture. It is likely that acupuncture alleviates ST-36 through regulating the disturbed glycerophospholipid metabolism, ether lipid metabolism, fatty acid metabolism, glycerolipid metabolism, porphyrin metabolism, sphingolipid metabolism and primary bile acid biosynthesis metabolism. Proteomics findings further extended our understanding on the protein changes related to transport, enzymatic activity, signaling pathway or receptor interaction *etc*, and may provide new clues for exploring the mechanisms of acupuncture on ST-36. The systems biology approach suggested that the acupuncture treatment involved in transport, signaling pathway, and receptor interaction. Our study also highlights the importance of systems biology as a potential tool to assess acupuncture treatment, eventually lead to the integration acupuncture with contemporary medicine. Although the study does examine an interesting application in alternative medicine, in any case, it would require a larger cohort design in order to derived greater statistical power. Next research on ST-36 includes the following three aspects: (1) to integrate the metabolomic and proteomic data sets as a way to further validate the biological significance of our preliminary findings; (2) the data needs to be re-evaluated when using other corrections to determine which metabolites remain significantly perturbed from baseline; (3) to certain enzymes associated with putative metabolite markers of treatment response differentially expressed within defined pathways.

Acupuncture is a traditional medical therapy that uses hair-thin metal needles to puncture the skin at specific points on the body to relieve pain and promote wellbeing that is used widely around the world. For World Health Organization proposed ‘Health for All’, we should be to promote the use of acupuncture medicine. It is an urgent need to develop new and innovative technologies to explore the underlying biological mechanisms of acupuncture. However, the exact mechanisms of acupuncture are currently unknown and it is likely that the body’s nervous system, neurotransmitters, and endogenous substances are involved in needle stimulation. Integrative multi-omics analysis in the post-genomic Era has been able to identify potential candidates for the effects of acupuncture and provides valuable information toward understanding mechanisms of therapy. In its grandest vision, the application of multi-omic systems approaches to the study of disease-perturbed networks will, through analyzing numerous metabolites, and could provides platform for research of the essence of acupuncture. It will pave the way for a better understanding of the mechanisms of acupuncture, ultimately advance the acupuncture medicine in the 21st century. We expect that in future, a multi-omic systems-based approach would serve as “Trojan horses” in the field of acupuncture field. With this development, it appears all the ancient concepts can be re-interpreted and harmonized with the integrative multi-omics analysis.

## Materials and Methods

### Clinical Study

The study was approved by the Ethics Committee at Heilongjiang University of Chinese Medicine (approval number: HUCM-CTRP-2012-108, date of registration is 09-10, 2012). Our study was designed in accordance with the ethical standards formulated in the Helsinki Declaration. The informed consent was obtained from all subjects. Additional experimental procedures are described in the Supporting Materials.

### In-Laboratory Session

Acupuncture stimulation was performed on bilateral ST-36 for 30 min, once a day for two weeks. Blood samples were collected from venous at 0 day (untreatment) and 14 day (treatment). Each blood sample (5 mL) was collected into the tube for 30 min and then centrifuged at 4 000 rpm for 10 min, then the serum sample was separated and stored at −80 °C until further analysis (SI Appendix, Materials and Methods).

### Metabolomic Analysis

Details of the untargeted and targeted metabolomics analysis are given in SI Appendix, Materials and Methods.

### iTRAQ proteome analysis

Details of the proteomics analysis as well as all statistical analyses, are given in SI Appendix, Materials and Methods.

## Additional Information

**How to cite this article**: Zhang, A. *et al.* Deciphering the biological effects of acupuncture treatment modulating multiple metabolism pathways. *Sci. Rep.* **6**, 19942; doi: 10.1038/srep19942 (2016).

## Supplementary Material

Supplementary Information
