# Supplementary material for: Deciphering the biological effects of acupuncture treatment modulating multiple metabolism pathways
Source: Sci Rep. 2016 Feb 16;6:19942. doi: 10.1038/srep19942 (PMC4754631; doi:10.1038/srep19942)
Supplement: Supplementary Information [file srep19942-s1.doc]

**Deciphering the biological effects of acupuncture treatment modulating multiple metabolism pathways**

Aihua Zhang, Guangli Yan, Hui Sun, Weiping Cheng, Xiangcai Meng, Li Liu, Ning Xie, Xijun Wang*

*National TCM Key Laboratory of Serum Pharmacochemistry, Sino-US Technology Cooperation Center of Chinmedomics, Research Center of Chinmedomics (State Administration of TCM), Laboratory of Metabolomics, Key Pharmacometabolomics Platform of Chinese Medicines, Department of Pharmaceutical Analysis, Heilongjiang University of Chinese Medicine, Heping Road, Harbin, China.*

* Address correspondence to:

Prof. Xijun Wang

National TCM Key Laboratory of Serum Pharmacochemistry

Sino-US Technology Cooperation Center of Chinmedomics

Research Center of Chinmedomics (State Administration of TCM)

Laboratory of Metabolomics

Key Pharmacometabolomics Platform of Chinese Medicines

Heilongjiang University of Chinese Medicine

Heping Road, Harbin, China.

Tel. & Fax + 0451-82110818

Email: xijunwangtcm@yeah.net

**Materials and Methods**

**Study design and cohort selection**

The informed consent was obtained from all subjects. The experimental procedures were approved by the Care and Ethics Committee at Heilongjiang University of Chinese Medicine (approval number: HUCM-CTRP-2012-108, date of registration is 09-10, 2012) and was conducted according to the principles expressed in the Declaration of Helsinki. Control healthy subjects (n = 20) with a mean age ± SD of 25.4 ± 4.2 years were enrolled in this study from Heilongjiang University of Chinese Medicine, China. In the First Affiliated Hospital, Heilongjiang University of Chinese Medicine, acupuncture stimulation was performed on bilateral ST-36 for 30 min (standardized acupuncture treatments), once a day for two weeks, due to two weeks as an optimum time period for acupuncture intervention. The outcomes of Health Survey Questionnaire were assessed, and the related clinical information was collected in Supplementary table 1. All young male healthy subjects were without underlying chronic disease (hypertension, type 2 diabetes, etc). Food (standard diet) and water (uniform labeled water) were available throughout the study, and the intake of alcohol and drug was forbidden during the study.

**Chemicals and reagents**

Acetonitrile (HPLC grade) was purchased from Merck (Germany), respectively; the distilled water was produced by a Milli-Q Ultra-pure water system (Millipore, Billerica, USA); formic acid (HPLC grade) was obtained from Kermel Chemical reagent Co., ltd. (China); leucine enkephalin was purchased from Sigma-Aldrich (St. Louis, MO, USA). All other reagents were of analytical grade.

**Sample preparation**

Blood samples were collected from venous at 0 day (untreatment) and 14 day (treatment). The control morning serum samples at 0 day were collected from every participant between 5:00 and 7:00 a.m before breakfast on day 1. Subsequently acupuncture stimulation was performed once a day for two weeks and individual morning serum samples (treatment) were collected from persons between 5:00 and 7:00 a.m before breakfast on day 14. Each blood sample (5 mL) was collected into the tube for 30 min and then centrifuged at 4 000 rpm for 10 min, then the serum sample was separated and stored at -80 °C until further analysis. Prior to UPLC-MS analysis, serum samples were thawed at room temperature. After vigorous shaking for 1 min and centrifuged 4 000 rpm for 10 min. Taking the upper serum (300μL) plus 4 times the amount (1200μL) mixed solution of acetonitrile - methanol - acetone (1:1:1) and centrifuged 12500 rpm for 10 mim. Taking the upper serum (1000μL) was evaporated under a stream of nitrogen, and the residue was dissolved in 200μL 80% methanol and centrifuged 13000 rpm for 15 mim. The supernatant (5μL) was used for UPLC-MS analysis. To ensure the stability and repeatability of the UPLC-MS system, each serum sample was mixed to generate a pooled quality control sample, which was treated using the above method and analyzed together with the real samples.

**Instrumentation and method configuration**

***Metabolomics analysis platform***

Chromatography was carried out with an ACQUITY BEH C_18_ column (100mm×2.1mm, 1.7um) using an UPLC^TM^ system (Waters Corp., Milford, USA). The column was maintained at 50 ºC, and subsequently, a gradient of 0.1% formic acid in acetonitrile (solvent A) and 0.1% formic acid in water (solvent B) used as follows: a linear gradient of 0–1.5 min, 5-15% A; 1.5–2.5 min, 15–35% A; 2.5–4.0 min, 35–45% A; 4.0–8.0 min, 45-65% A; 8–10 min, 65–99% A; 10–11 min, 99% A. The ﬂow rate was 0.50 mL/min and 2 μL aliquot of each sample was injected onto the column. The eluent was introduced into the Synapt^TM^ High Definition MS (Waters Corp., Milford, USA) analysis, and the optimal conditions were as follows: desolvation temperature of 350 ºC, source temperature of 110 ºC, cone gas flow of 50 L/h and desolvation gas flow of 700 L/h, sample cone voltage of 30V, extraction cone voltage of 3.5V for positive ion mode and 4.0V negative ion mode, collision energy of 4 eV, microchannel plate voltage of 2400V, capillary voltage of 3.2 kV for positive ion mode and 2.6 kV negative ion mode. Leucine enkephalin at a concentration of 200 pg/mL was used via a lock spray interface at a ﬂowrate of 100 μl/min monitoring for positive ion mode ([M+H]^+^=556.2771) and negative ion mode ([M-H]^−^ = 554.2615) to ensure accuracy during the MS analysis. Data were collected in centroid mode and the mass range was set at m/z 100–1100 using extended dynamic range. This study typically include intermittent quality controls (pooled samples analyzed intermittently) as a way to estimate system drift and ensure adequate method robustness.

Mass spectrometric data matrix was processed and normalized using MassLynx V4.1 and MarkerLynx software (Waters Corp., Milford, USA). Multivariate data matrix was analyzed by EZinfo software (Waters Corp., Milford, USA). The unsupervised segregation was checked by principal components analysis (PCA), potential markers of interest were extracted from S-plots constructed following analysis with orthogonal partial least-squares to latent structures discriminant analysis (OPLS-DA). Potential metabolite markers were extracted using a statistically significant threshold of the variable importance in the projection (VIP>1) constructed from the PLS-DA analysis, where the markers were chosen based on their contributions to the variation and correlation within the data set, and two-tailed Student’s *t*-test on the normalized raw data. Metabolic pathway analysis was performed by MetaboAnalyst tool (http://www.metaboanalyst.ca) based on the database sources to identify the affected metabolic pathways and facilitate further biological interpretation.

***iTRAQ proteome analysis***

Labeling of samples with iTRAQ reagents was carried out according to the manufacturer’s instructions (iTRAQ Reagents Multiplex kit; Applied Biosystems/MDS Sciex, Foster City, CA). Briefly, 90 mg of pooled control or treated-acupuncture serum protein sample was reconstituted in dissolution buffer, denatured, reduced, alkylated and then trypsinized (1ug/ul trypsin; Promega, Madison, USA) for 24 h at 37°C. Tryptic digests were labeled with 8 different iTRAQ reagents.

Nanoflow electrospray ionization tandem mass spectrometric analysis of peptide samples was carried out using LTQ-Orbitrap Velos (Thermo Scientific, Bremen, Germany) interfaced with Agilent’s 1200 Series nanoflow LC system. The chromatographic capillary columns used were packed with Magic C_18_ (particle size 5 um, pore size 300A°; Michrom Bioresources, Auburn, CA, USA) reversed phase material in 99.9% ACN at a flow rate of 400nl/min. The peptides were eluted using a linear gradient (5% at 0 min 10% 2 min, 50% 42 min 80% 42–50 min, 5% 50–60 min) over 60 min. Mass spectrometric analysis was carried out in a data dependent manner with 350-2000Da scans acquired using the Orbitrap mass analyzer. The fragmentation was carried out using higher-energy collision dissociation. For accurate mass measurements, the lock mass option was enabled.

The resulting MS/MS spectra were searched with MASCOT software (Matrix Science, London, U.K.; version 2.3.01). For protein identification and quantification, a peptide mass tolerance of 20 ppm was allowed for intact peptide masses and 0.05 Da for fragmented ions. One missed cleavage was allowed in the trypsin digests; carbamidomethyl of cysteine was considered a fixed modification. All identified peptides had an ion score above the Mascot peptide identity threshold, and a protein was considered identified if at least one such unique peptide match was apparent for the protein. For protein-abundance ratios measured using iTRAQ, we set a 1.2-fold change as the threshold and a two-tailed p-value<0.05 to identify significant changes. Gene Ontology (GO) functional classifications were analyzed with Blast2GO software (http://www.blast2go.org/), and GO enrichment analysis was performed to identify GO terms that were significantly enriched in differentially expressed proteins. Target validation of proteomic results was performed on the serum biosamples. We validated the expression of these proteins using Western blotting method.

**Statistical Analysis**

All statistical analyses were performed using the Student’s t-test. Differences with a P-value of 0.05 or less were considered signiﬁcant. Bonferroni-corrected p-values are recommended to avoid bias and false discovery. The false discovery rate was used to correct for the multiple testing problem.

**
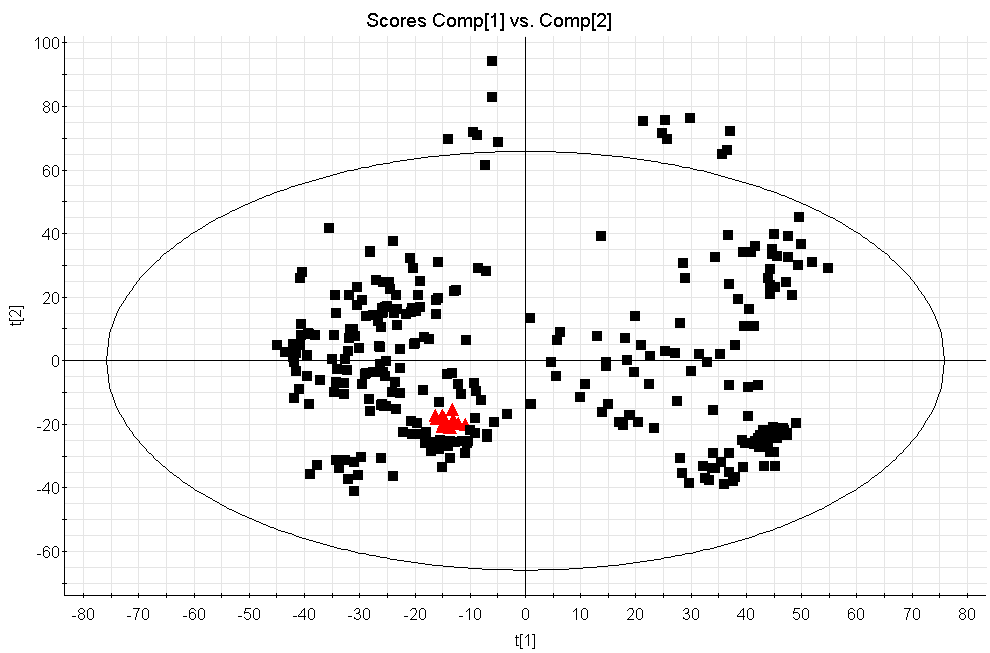
**

**Fig. S1.** Principal component analysis of QC urine samples carried out with LC/MS.

Black box present urine samples, and samples in red box present QCs.

**Supplementary Table 1**. Clinical characteristics of the male subjects at baseline

| **Samples** | **Subjects** |
| --- | --- |
| Sample No. | 20 |
| Age | 25.4 ± 4.2 |
| BMI(kg/m2) | 6/6 |
| ALT (U/L) | 21.59±2.38 |
| AST (U/L) | 64.84±19.78 |
| Total bilirubin (mg/dL) | 88.49±42.09 |
| Direct bilirubin(mg/dL) | 2.09±1.42 |
| Indirect bilirubin(mg/dL) | 2.84 ± 1.08 |

**Supplementary table 2.** Potential differentiating metabolites identified in acupuncture-treated human.

| **No.** | **Rt(min)** | **m/z determined** | **m/z calculated** | **Error(mDa)** | **Ion form** | **Formula** | **Metabolite Name** | **VIP Value** | **Trend** | **P-value** |
| --- | --- | --- | --- | --- | --- | --- | --- | --- | --- | --- |
| 1 | 0.47 | 280.0916 | 280.0926 | -1.0 | [M+Na]^+^ | C_8_H_20_NO_6_P | Glycerophosphocholine | 4.6 | ↑ | 5.12E-03 |
| 2 | 0.52 | 258.1121 | 258.1107 | 1.4 | [M+H]^+^ | C_8_H_20_NO_6_P | sn-glycero-3-Phosphocholine | 1.3 | ↑ | 7.81E-03 |
| 3 | 4.62 | 274.2743 | 274.2746 | -0.3 | [M+H]^+^ | C_16_H_35_NO_2_ | Sphinganine | 10.9 | ↓ | 2.39E-03 |
| 4 | 4.66 | 230.2475 | 230.2484 | -0.9 | [M+H]^+^ | C_27_H_46_O | 2-amino-tridecanoic acid | 1.6 | ↓ | 2.07E-02 |
| 5 | 7.42 | 520.3392 | 520.3403 | -1.1 | [M+H]^+^ | C_26_H_50_NO_7_P | LysoPC(18:2(9Z,12Z)) | 16.3 | ↑ | 2.17E-03 |
| 6 | 8.41 | 480.3439 | 480.3454 | -1.5 | [M+H]^+^ | C_24_H_50_NO_6_P | LysoPC(P-16:0) | 5.0 | ↑ | 7.11E-03 |
| 7 | 8.76 | 506.3597 | 506.3611 | -1.4 | [M+H]^+^ | C_26_H_52_NO_6_P | LysoPC(P-18:1(9Z)) | 1.7 | ↑ | 1.78E-02 |
| 8 | 9.48 | 374.3051 | 374.3035 | 1.6 | [M+Na]^+^ | C_22_H_41_NO_2_ | Anandamide (20:2, n-6) | 6.2 | ↓ | 1.38E-04 |
| 9 | 9.53 | 508.3767 | 508.3767 | 0.0 | [M+H]^+^ | C_26_H_54_NO_6_P | LysoPC(P-18:0/0:0) | 2.0 | ↑ | 3.26E-02 |
| 10 | 9.55 | 510.3907 | 510.3924 | -1.7 | [M+H]^+^ | C_26_H_56_NO_6_P | LysoPC(O-18:0) | 2.9 | ↑ | 1.15E-02 |
| 11 | 9.89 | 780.5506 | 780.5519 | -1.3 | [M+Na]^+^ | C_42_H_80_NO_8_P | PC 34:2 | 3.5 | ↑ | 3.85E-04 |
| 12 | 9.89 | 808.5820 | 808.5832 | -1.2 | [M+Na]^+^ | C_44_H_84_NO_8_P | PC 36:2 | 2.8 | ↑ | 4.79E-04 |
| 13* | 9.90 | 336.3234 | 336.3242 | -0.8 | [M+Na]^+^ | C_20_H_43_NO | 2-amino-14,16-dimethyloctadecan-3-ol | 3.3 | ↓ | 3.09E-05 |
| 14 | 9.90 | 725.5596 | 725.5614 | -1.8 | [M+Na]^+^ | C_39_H_79_N_2_O_6_P | SM(d18:0/16:1(9Z)) | 1.6 | ↑ | 2.92E-03 |
| 15 | 9.91 | 353.2657 | 353.2668 | -1.1 | [M+Na]^+^ | C_19_H_38_O_4_ | MG(16:0/0:0/0:0) | 4.2 | ↓ | 2.17E-04 |
| 16 | 10.17 | 433.3660 | 433.3658 | 0.2 | [M+Na]^+^ | C_26_H_50_O_3_ | 3-oxohexacosanoic acid | 1.3 | ↓ | 2.63E-02 |
| 17 | 10.22 | 780.5521 | 780.5519 | 0.2 | [M+Na]^+^ | C_42_H_80_NO_8_P | PC 34:2 | 2.0 | ↑ | 9.52E-03 |
| 18 | 10.23 | 808.5811 | 808.5832 | -2.1 | [M+Na]^+^ | C_44_H_84_NO_8_P | PC 36:2 | 4.4 | ↑ | 1.43E-02 |
| 19 | 10.27 | 463.3760 | 463.3763 | -0.3 | [M+Na]^+^ | C_27_H_52_O_4_ | MG(24:1(15Z)/0:0/0:0) | 1.4 | ↓ | 1.94E-02 |
| 20 | 10.45 | 381.2978 | 381.2981 | -0.3 | [M+Na]^+^ | C_21_H_42_O_4_ | MG(18:0/0:0/0:0) | 1.2 | ↓ | 2.84E-03 |
| 21 | 1.83 | 212.0014 | 212.0018 | -0.4 | [M-H]^-^ | C_8_H_7_NO_4_S | Indoxyl sulfate | 2.4 | ↓ | 4.32E-02 |
| 22 | 6.52 | 452.2758 | 452.2777 | -1.9 | [M-CH3]^-^ | C_22_H_46_NO_7_P | LysoPC(14:0) | 1.4 | ↓ | 2.98E-03 |
| 23 | 7.35 | 524.2776 | 524.2774 | 0.2 | [M-H]^-^ | C_27_H_44_NO_7_P | LysoPE(22:6(4Z,7Z,10Z,13Z,16Z,19Z)/0:0) | 1.5 | ↓ | 1.86E-02 |
| 24 | 7.39 | 476.2774 | 476.2777 | -0.3 | [M-H]^-^ | C_23_H_44_NO_7_P | LysoPE(18:2(9Z,12Z)/0:0) | 3.2 | ↓ | 7.82E-03 |
| 25 | 7.40 | 500.2760 | 500.2777 | -1.7 | [M-H]^-^ | C_25_H_44_NO_7_P | LysoPE(20:4(8Z,11Z,14Z,17Z)/0:0) | 1.7 | ↓ | 3.22E-02 |
| 26 | 7.72 | 540.3296 | 540.3301 | -0.5 | [M+HCOO]^-^ | C_24_H_50_NO_7_P | LysoPC(0:0/16:0) | 3.6 | ↓ | 1.36E-02 |
| 27 | 8.03 | 480.3059 | 480.3090 | -3.1 | [M-CH3]^-^ | C_24_H_50_NO_7_P | LysoPC(16:0/0:0) | 4.5 | ↓ | 2.56E-02 |
| 28 | 8.36 | 478.2923 | 478.2934 | -1.1 | [M-H]^-^ | C_23_H_46_NO_7_P | LysoPE(18:1(11Z)/0:0) | 1.5 | ↓ | 1.45E-02 |
| 29 | 8.40 | 436.222 | 436.2828 | 0.1 | [M-H]^-^ | C_21_H_44_NO_6_P | LysoPE(P-16:0/0:0) | 2.0 | ↑ | 2.44E-02 |
| 30 | 8.41 | 524.3356 | 524.3352 | 0.4 | [M+HCOO]^-^ | C_24_H_50_NO_6_P | LysoPC(P-16:0) | 2.5 | ↑ | 1.42E-03 |
| 31 | 8.74 | 462.2987 | 462.2984 | 0.3 | [M-H]^-^ | C_23_H_46_NO_6_P | LysoPE(17:2) | 1.6 | ↑ | 4.23E-02 |
| 32 | 8.77 | 552.3661 | 552.3665 | -0.4 | [M+HCOO]^-^ | C_26_H_54_NO_6_P | LysoPC(0:0/P-18:0) | 1.3 | ↑ | 4.84E-02 |
| 33 | 8.78 | 554.3471 | 554.3458 | 1.3 | [M+HCOO]^-^ | C_25_H_52_NO_7_P | LysoPC(17:0) | 1.2 | ↓ | 3.32E-02 |
| 34 | 9.32 | 508.3407 | 508.3403 | 0.4 | [M-CH3]^-^ | C_26_H_54_NO_7_P | LysoPC(18:0) | 3.0 | ↓ | 1.02E-02 |

Note: * Both in positive and negative mode.

**Supplementary table 3**. Ingenuity pathway analysis result for metabolomics data.

| No. | Pathway Name | Total | Expected | Hits | Raw p | Impact |
| --- | --- | --- | --- | --- | --- | --- |
| 1 | Glycerophospholipid metabolism | 39 | 0.17823 | 3 | 0.000594 | 0.11414 |
| 2 | Ether lipid metabolism | 23 | 0.10511 | 2 | 0.00456 | 0.04204 |
| 3 | Fatty acid metabolism | 50 | 0.2285 | 1 | 0.20657 | 0.02959 |
| 4 | Glycerolipid metabolism | 32 | 0.14624 | 1 | 0.13716 | 0.01247 |
| 5 | Porphyrin and chlorophyll metabolism | 104 | 0.47528 | 1 | 0.38546 | 0.01101 |
| 6 | Sphingolipid metabolism | 25 | 0.11425 | 1 | 0.10871 | 0.00954 |
| 7 | Primary bile acid biosynthesis | 47 | 0.21479 | 1 | 0.19537 | 0.00537 |
| 8 | Fatty acid elongation in mitochondria | 27 | 0.12339 | 1 | 0.11693 | 0 |
| 9 | Fatty acid biosynthesis | 49 | 0.22393 | 1 | 0.20285 | 0 |
| 10 | Tryptophan metabolism | 79 | 0.36103 | 1 | 0.30779 | 0 |

Note: Total is the total number of compounds in the pathway; the Hits is the actually matched number from the user uploaded data; the Raw p is the original p value calculated from the enrichment analysis; the Impact is the pathway impact value calculated from pathway topology analysis.

**Supplementary table 4**. List of differentially expressed proteins identified by MALDI-TOF-MS/MS analysis.

| **No.** | **Accession(gi)** | **Description** | **Score** | **Coverage** | **# Proteins** | **# Unique Peptides** | **# Peptides** | **# PSMs** | **# AAs** | **MW [kDa]** | **calc. pI** | **Treatment/control** |
| --- | --- | --- | --- | --- | --- | --- | --- | --- | --- | --- | --- | --- |
| 1 | 178804 | apolipoprotein B-100 precursor | 23581.34 | 61.17% | 30 | 9 | 242 | 3035 | 4563 | 515.3 | 6.99 | 0.656 |
| 2 | 62087272 | Apolipoprotein B variant | 9584.49 | 59.13% | 7 | 1 | 82 | 1109 | 1615 | 183.5 | 6.8 | 0.727 |
| 3 | 28590 | unnamed protein product | 8526.68 | 75.70% | 3 | 1 | 47 | 1296 | 609 | 69.3 | 6.28 | 1.202 |
| 4 | 23307793 | serum albumin | 8041.57 | 74.06% | 12 | 2 | 47 | 1377 | 609 | 69.3 | 6.54 | 1.302 |
| 5 | 12054080 | immunoglobulin heavy chain constant region mu | 6556.93 | 61.59% | 8 | 1 | 20 | 942 | 453 | 49.4 | 6.83 | 1.391 |
| 6 | 53791223 | fibronectin 1 | 6229.85 | 49.05% | 53 | 1 | 72 | 840 | 2265 | 249.3 | 5.99 | 0.538 |
| 7 | 119625326 | fibrinogen gamma chain, isoform CRA_o | 4687.39 | 63.81% | 19 | 3 | 27 | 920 | 420 | 47.3 | 5.85 | 0.724 |
| 8 | 119625310 | fibrinogen gamma chain, isoform CRA_a | 3830.34 | 66.77% | 7 | 1 | 25 | 756 | 334 | 37.7 | 6.29 | 0.807 |
| 9 | 27597169 | FGA | 2802.23 | 73.77% | 1 | 1 | 5 | 264 | 61 | 7 | 8.5 | 1.216 |
| 10 | 119573007 | apolipoprotein A-II, isoform CRA_d | 2794.66 | 75.00% | 4 | 2 | 8 | 469 | 92 | 10.6 | 9.04 | 0.812 |
| 11 | 58032609 | anti-pneumococcal antibody 57E2 light chain | 2780.88 | 47.40% | 84 | 1 | 6 | 432 | 192 | 20.9 | 7.77 | 0.825 |
| 12 | 34527666 | unnamed protein product | 2509.46 | 42.22% | 217 | 1 | 15 | 393 | 469 | 50.9 | 8.43 | 1.748 |
| 13 | 21669349 | immunoglobulin kappa light chain VLJ region | 2243.83 | 40.89% | 738 | 1 | 7 | 400 | 269 | 28.9 | 7.14 | 1.209 |
| 14 | 21669339 | immunoglobulin kappa light chain VLJ region | 2086.16 | 33.33% | 26 | 1 | 6 | 381 | 270 | 29.3 | 6.67 | 1.309 |
| 15 | 178741 | apolipoprotein C-II | 1562.34 | 58.24% | 6 | 5 | 5 | 245 | 91 | 10.2 | 4.56 | 0.809 |
| 16 | 215982752 | immunoglobulin kappa light chain variable region | 1393.49 | 46.38% | 537 | 1 | 4 | 234 | 138 | 14.7 | 8.62 | 1.747 |
| 17 | 119596192 | phospholipid transfer protein, isoform CRA_d | 1351.48 | 49.35% | 9 | 16 | 16 | 94 | 462 | 51.5 | 6.55 | 0.78 |
| 18 | 34526163 | unnamed protein product | 1285.45 | 41.80% | 5 | 1 | 15 | 152 | 512 | 55.5 | 7.31 | 0.804 |
| 19 | 297264957 | PREDICTED: tubulin alpha-4A chain | 1184.52 | 42.49% | 20 | 4 | 13 | 70 | 433 | 48.3 | 5.01 | 0.823 |
| 20 | 498894 | unnamed protein product | 1140.46 | 15.56% | 5 | 3 | 3 | 140 | 225 | 25.3 | 9.54 | 0.579 |
| 21 | 194383496 | unnamed protein product | 1117.29 | 55.70% | 15 | 26 | 26 | 112 | 605 | 68.3 | 5.82 | 1.438 |
| 22 | 567112 | OMM protein (Ig gamma3) heavy chain | 1095.53 | 40.89% | 3 | 1 | 11 | 167 | 313 | 34.9 | 7.49 | 0.814 |
